# Metabolomic profiling of biphenyl-induced stress response of *Brucella anthropi* MAPB-9

**DOI:** 10.1038/s41598-025-95867-1

**Published:** 2025-04-05

**Authors:** Monika Sandhu, Atish T. Paul, Prabhat N. Jha

**Affiliations:** 1 Department of Biological Sciences, Birla Institute of Technology and Science Pilani, Pilani campus, Pilani, Rajasthan 333031 India; 2 Department of Pharmacy, Birla Institute of Technology and Science Pilani, Pilani campus, Pilani, Rajasthan 333031 India

**Keywords:** Metabolomic, Biodegradation, Polychlorinated biphenyl, Nutritional stress, *Ochrobactrum anthropi*, *Brucella anthropi*, Microbiology, Environmental microbiology, Soil microbiology

## Abstract

The exposure of bacteria to toxic compounds such as polychlorinated biphenyl (PCB) and biphenyl induces an adaptive response at different levels of cell morphology, biochemistry, and physiology. PCB and biphenyl are highly toxic compounds commercially used in the industry. In our previous study, *Brucella anthropi* MAPB-9 efficiently degraded PCB-77 and biphenyl at a high concentration. In this study, we used metabolomic analyses to understand the metabolic processes occurring in MAPB-9 during exposure to biphenyl. A combination of analytical techniques such as GC-MS/MS and HR-MS study confirmed the complete biphenyl degradation pathway. The intermediate metabolic products identified were cis-2, 3-dihydro-2, 3-dihydroxy biphenyl, 2,3-dihydroxy biphenyl, and 4-dihydroxy-2-oxo-valerate. Further, benzoic acid and 2,3-dihydroxy benzoic acid metabolites identified in the extract revealed the interconnection of biphenyl and benzoic degradation pathways. In addition, the variations in the functioning of the major biochemical pathways in the cells were revealed through changes in the profile of metabolites belonging to glyoxylate, tricarboxylic acid (TCA) cycle, and fatty acid pathways. The exposure to biphenyl inhibited metabolic activity leading to changes in the morphology and metabolism. Despite many adverse changes, the MAPB-9 was able to adapt and grow in the toxic environment undergoing upper and lower biphenyl degradation pathways.

## Introduction

Polychlorinated biphenyl (PCB) and biphenyl are well-known persistent organic pollutants that adversely affect the environment and human health. Bioremediation is a promising alternative to other conventional physical and chemical methods for the degradation of contaminants like PCB. The use of microorganisms capable of utilizing organic contaminants as a carbon source is the essence of bioremediation. Some microorganisms survive in the contaminated site by degrading the contaminant using an enzyme or cofactor during the oxidation or reduction of carbon-containing complex organic compounds. The complete biphenyl catabolic pathway includes the upper and lower biodegradation pathways^1^. The biphenyl-degrading bacteria lack a complete catabolic pathway^2^, which results in the accumulation of dead-end products, thereby inhibiting the growth of bacterial cells during the biodegradation process^3^. Benzoic acid and chlorobenzoic acid (CBA) have been identified as dead-end intermediates^4^ produced during biphenyl degradation. However, it could further be transformed or oxidized by other bacteria that possess benzoate catabolic enzymes^5^. Therefore, identifying the metabolites formed during the biodegradation process is very important.

Metabolites are small, low-molecular-weight biological molecules that play an important role in energy conversion and metabolic pathways. The investigation of the entire set of intra and extracellular metabolites, the small molecule substrates, intermediates, products of the cell metabolism, extracted from proliferating bacterial cells at a specific time of their growth phase directly reflecting the outcome of complex networks of biochemical reactions, is called metabolomics. Metabolomics provides insights into multiple aspects of cellular physiology with ‘omics’ sciences that utilize a couple of analytical tools. However, conventional methods of analysis of metabolites are extremely challenging due to the long time required for sample preparation, a wide concentration range, and optimization of the conditions for analysis. Also, the limited number of sample injections, slow sample analysis, and data processing results in low throughput rates. The invention of modern hyphenated analytical chromatographic platforms such as Gas and Liquid chromatography coupled with mass spectrometry resulted in quick sampling and quenching of cellular activity, along with fast extraction and analysis of the metabolites. Metabolite profiling employing an untargeted approach can be used to identify metabolites/intermediates produced during the metabolic degradation of PCB/biphenyl.

It determines the total concentration or relative level of the metabolites that accumulate in the predefined group, such as organic acids, amino acids, and carbohydrates. In contrast, targeted metabolite analysis determines the profiling of the specific metabolites involved in a particular pathway. Metabolite analysis requires specialized extraction procedures, separation techniques, and specific detection methods. GC-MS/MS is the preferred technique for separating low molecular weight metabolites that are either volatile or can be converted into volatile compounds through chemical derivatization before analysis. Derivatization enhances volatility and reduces the polarity of carboxyl (-COOH), amine (-NH_2_), thiol (-SH), and polar hydroxyl (-OH) groups^6^.

PCB-degraded metabolites include the byproducts and intermediates of biphenyl/PCB catabolism. Moreover, some of the metabolites are also exclusively produced in response to stress induced by biphenyls or PCBs. To combat these pollutant stresses, bacteria use different defense strategies like morphological and physiological modification and increased production of shock proteins DnaK and GroEL. The effect of nutritional stress on growth behaviors and metabolomic profiles of biphenyl/PCB-degrading bacteria such as *Pseudomonas* and *Burkholderia* has been reported in a couple of studies^7,8^. However, metabolome level changes, particularly in bacteria capable of degrading PCB and biphenyl, are still poorly understood. These studies are limited to the targeted metabolite identification involved in particular metabolic pathways in bacterial cells exposed to biphenyl/PCB. Hence, the analysis of untargeted and targeted metabolomics profiles concerning biphenyl/PCB uptake, catabolism, and cellular response will be instrumental in understanding the biphenyl/PCB remediation by bacteria.

In our previous study, *Brucella anthropi* MAPB-9 was reported to be isolated from PCB-contaminated nearby soil of the Bhilai Steel plant, Chattisgarh, India^9^. *B. anthropi* MAPB-9 exhibited the potential to degrade biphenyl and PCB-77, 100% and 30.49%, respectively^10^. Some reports highlight the degradation potential of *B. anthropi* towards PCB congeners^8^. Therefore, the present study aimed to identify PCB-induced metabolites (untargeted) and intermediates of PCB-catabolic pathway (targeted) produced in *B. antrhopi* MAPB-9. We employed gas chromatography-mass spectrometry (GC-MS) based metabolomics to identify the underlying metabolic signatures and their role in biphenyl degradation and adaptation mechanisms. The profiling of metabolites and identification of the metabolic pathways involved under biphenyl-induced stress resulted in an understanding the adaptive mechanism of the isolate *Brucella anthropi* MAPB-9. To our knowledge, this study is the first to investigate the metabolomics profile of *B. anthropi* using a GC-MS/MS-based metabolomics approach and its adaptive strategy to combat stress. Biodegradation potential and the adaptive mechanism of MAPB-9 under biphenyl-induced stress conditions could be further explored for strain improvement to remediate persistent organic pollutants in the soil.

## Materials and methods

### Chemicals and reagents

Chemicals and reagents of the commercially available highest grade were used. Biphenyl, ethyl acetate, methanol, acetonitrile, formic acid, TCMS (Trimethylchlorosilane), and BSTFA (N, O-bis(trimethylsilyl) trifluoroacetamide) were purchased from Sigma-Aldrich (Germany).

### Bacterial culture and growth conditions of *B. anthropi* MAPB-9

Isolate *B. anthropi* MAPB-9 was isolated from a PCB-contaminated site in our previous study^9^ and was used in the study. Experiments were carried out to investigate cell morphology and metabolomics involved in biphenyl degradation by MAPB-9.

### Growth conditions of MAPB-9 bacterial cell on biphenyl

In an incubator-shaker, isolate MAPB-9 was grown in Luria Broth to the late log phase (OD_600_ 0.8) at 30 °C, 150 rpm. After incubation, 5 ml suspension was aseptically transferred to a 15 mL falcon and centrifuged at 8000 ×g for 10 min. The cell pellet was collected and washed twice with Minimal Media (MM). The pellet was resuspended in 1 mL of MM to be used as the inoculum. A 0.5 mL cell suspension was added to 4.5 mL of MM supplemented with biphenyl (200 mg L^− 1^) and was incubated at 30 °C, 150 rpm in a shaker for 72 h in 30 mL screw-capped vials. After incubation, the cellular morphology and metabolomic profile of the isolate grown in a biphenyl-supplemented medium were examined. Control was set as MM supplemented with 0.2% glucose inoculated with the selected isolate.

### Morphological analyses of MAPB-9 by field emission scanning electron microscopy (FESEM)

The FESEM analysis of the isolate MAPB-9 was performed to observe the effect of biphenyl on the cell morphology. The bacterial cells were grown in LB and MM supplemented with biphenyl 200 mg L^− 1^ in the presence of 0.2% glucose and incubated for 72 h at 30 °C to examine the cell morphology. After incubation, the smear was prepared on the glass slide and fixed with 2.5% (*v/v*) glutaraldehyde solution for an hour. The fixed bacterial cells were then subjected to gradual ethanol dehydration, i.e., 50–100% (*v/v*), with 10 min intervals each for drying. The gold sputter coating was done to the fixed bacterial cells for 30 s. The bacterial cells were observed under 20 kV FESEM (FEI™ Thermo Fischer Scientific, Apreo) at different magnification powers.

### Metabolomic analysis of MAPB-9 by GC-MS/MS and HRMS

*Extraction and derivatization of metabolites produced in biphenyl-supplemented MM*.

After incubation, the sample was extracted to identify the metabolites produced during biphenyl degradation. The cultivation media was acidified with HCl (6 N) to attain a pH of 2, and the sample was extracted three times with ethyl acetate. The ethyl acetate was collected and evaporated by a rotary evaporator.

The extracts were derivatized with 100 µL of bis-(trimethylsilyl) trifluoroacetamide (BSTFA) at 37 °C for 60 min and with trimethylchlorosilane (TMCS) (99:1, *v/v*) at 60 °C for 15 min^11^ and were analyzed by GC-MS/MS TQ8040 (Shimadzu, Japan) in Scan and MRM (Multiple reaction monitoring) mode.

### GC-MS/MS condition for the separation of the metabolites produced in biphenyl-supplemented MM

The separation of metabolites produced in biphenyl-supplemented MM was performed on RXi-5SilMS fused silica column with dimensions of 30 m × 0.32 mm × 0.25 μm. The column temperature was programmed from 50 °C (2 min hold) to 80 °C at 10 °C/min (5 min hold), 100 °C rise at 5 °C/min (1 min hold), and finally to 300 °C at 15 °C/min (10 min hold). The carrier gas was helium, and the column flow rate was 1mL/min. The injection and the detector temperatures were 280 °C and 300 °C, respectively. Compounds separated by gas chromatography were detected by MS operating in full scan mode from *m/z* 45 to 500 at a scan rate of 1.68 scans.s^–1^. Metabolites were identified by the mass spectral NIST14 library provided with GC-MS/MS TQ8040. In GS-MS/MS MRM mode, the analysis was done by providing the retention time and collision energy.

### HR-MS of intermediate products produced in biphenyl-supplemented MM

In addition to the GC-MS/MS analysis, HRMS analysis was performed to determine the polar metabolite produced by MAPB-9 in the biphenyl-supplemented MM. The extract obtained after evaporating the solvent was further dissolved in DMSO for HR-MS analysis using Agilent Technologies 6545 Q-TOF LC/MS (Santa Clara, US). Detection conditions were as follows: electrospray ionization (ESI) (positive), isocratic conditions, and methanol: water (40:60; v/v). Some of the polar metabolites involved in the biphenyl degrading pathway by MAPB-9 were identified by Agilent mass hunter software by peak abundance to charge to mass (m/z).

### Metabolite set enrichment analysis

GC-MS/MS solution software was used to identify the metabolite produced by MAPB-9 grown in biphenyl-supplemented MM. MetaboAnalyst 5.0 online software analysis of the metabolites was used to map the pathway. A metabolite set enrichment analysis was performed to identify the active metabolism in the biphenyl-grown bacterial cell. Over-representation analysis (ORA) pathway approach was used for the functional interpretation of metabolomics datasets. To perform ORA, a list of differentially abundant metabolites was used as input, and Kyoto Encyclopedia of Genes and Genomes (KEGG) Pathway was used as a reference database for metabolic pathway enrichment^12,13^. The metabolite identified with GC-MS/MS was converted into a KEGG identifier and was used as input for analyzing the metabolite by MetaboAnalyst 5.0 an online software for metabolomic data. For enrichment analysis with well-annotated KEGG ID compounds (i.e., those in pathway libraries & metabolite sets) of the metabolic pathway in treated and control groups, was mapped.

### Statistical analysis

All the experiments were done in triplicates, representing data in ± standard error mean. The one-tailed test was performed, and a p-value below 5% is considered a statistically significant result. P-values for each pathway were calculated using a right-tailed Fisher’s exact test based on the hypergeometric distribution.

## Results and discussion

### FESEM analysis of cellular morphology of MAPB-9 in the presence of biphenyl

**Fig. 1:**
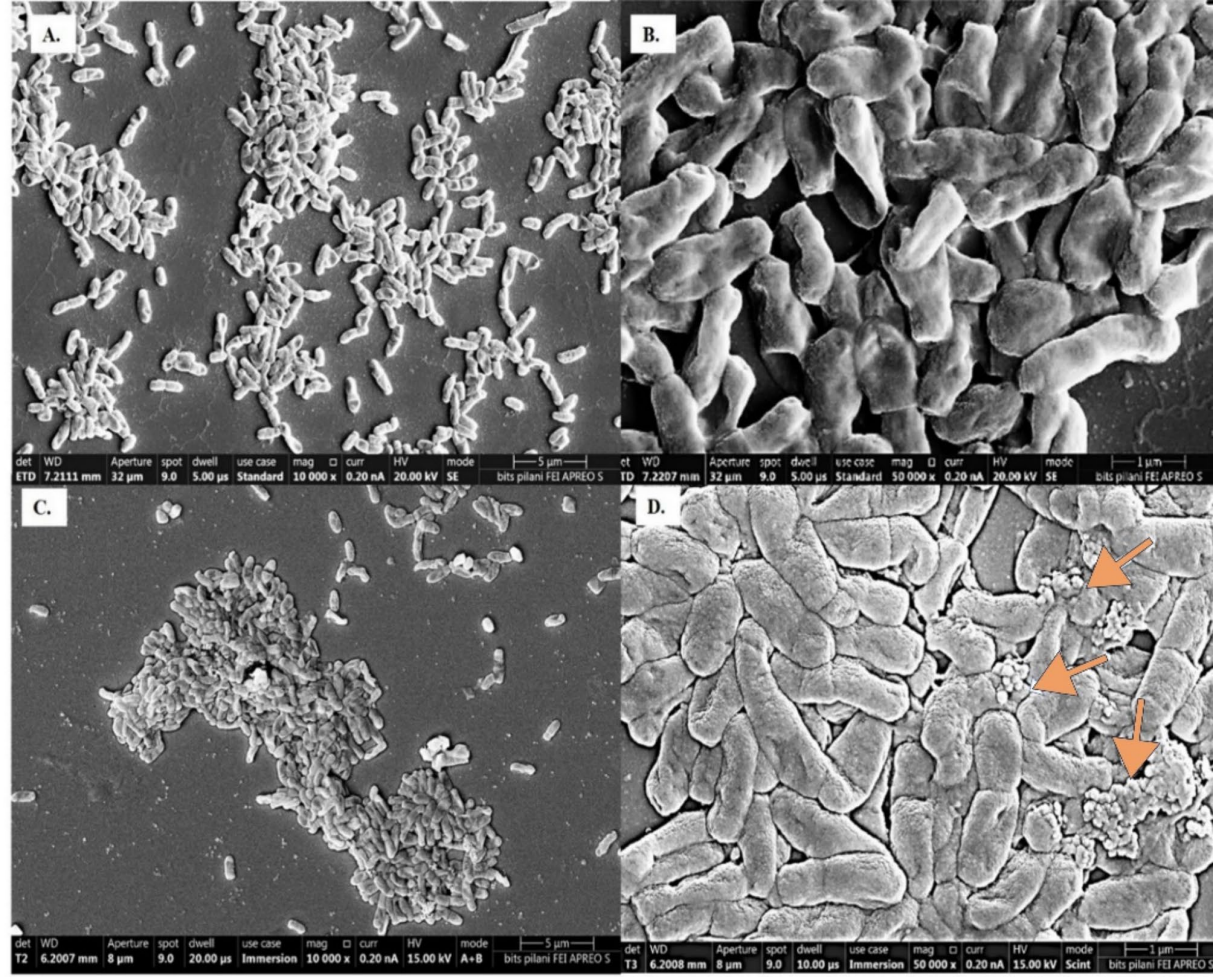
FESEM analysis of MAPB-9 grown in (**A**) LB media, 10,000X, 5 μm (**B**) LB media, 50000X, 1 μm and (**C**) MM with biphenyl, 10,000X, 5 μm (**D**) MM with biphenyl, 50,000 × 1 μm. Arrow indicates outer membrane vesicles.

The effect of biphenyl on the cellular morphology of MAPB-9 was analyzed by FESEM. The bacterial isolates showed normal morphology in the LB medium. MAPB-9 cells showed an assemblage pattern with a packed arrangement in a layer of bacterial cells lying one above another (Fig. 1A-D). Cell size decreased when grown in MM supplemented with biphenyl (Fig. 1 C) compared to cells grown under a nutrient-rich LB medium (Fig. 1A). The average cell size (*n* = 3) was found to be 1.748 ± 0.01 μm when grown in LB medium, while it was observed to be 1.563 ± 0.02 μm, in biphenyl-treated conditions.

In addition to the reduced size, the biphenyl-treated cells also showed increased secretion of exopolysaccharide layer (EPS) and formation of membrane vesicle-like structure (Fig. 1C,D). Similar to our observation, *Pseudomonas* sp. IH-2000^14^ and *P. putida* DOT-T1E^15^ produced membrane vesicles when toluene was added to the culture medium. The above study suggested that vesicle formation is a specific response of bacteria to remove toxic compounds. These physiological observations were also in accordance with changes in *Burkholderia xenovorans* LB400 during early stationary-phase biphenyl-grown cells. Due to exposure to organic pollutants such as biphenyl, PCBs, and toluene, membrane separation, size reduction, and formation of vesicles in a bacterial cell often indicate stress conditions^16^.

### Metabolomics analysis of MAPB-9 in biphenyl-supplemented MM revealved complete mineralization of the biphenyl

#### Identification of upper biphenyl and lower benzoate degradation metabolites

**Fig. 2:**
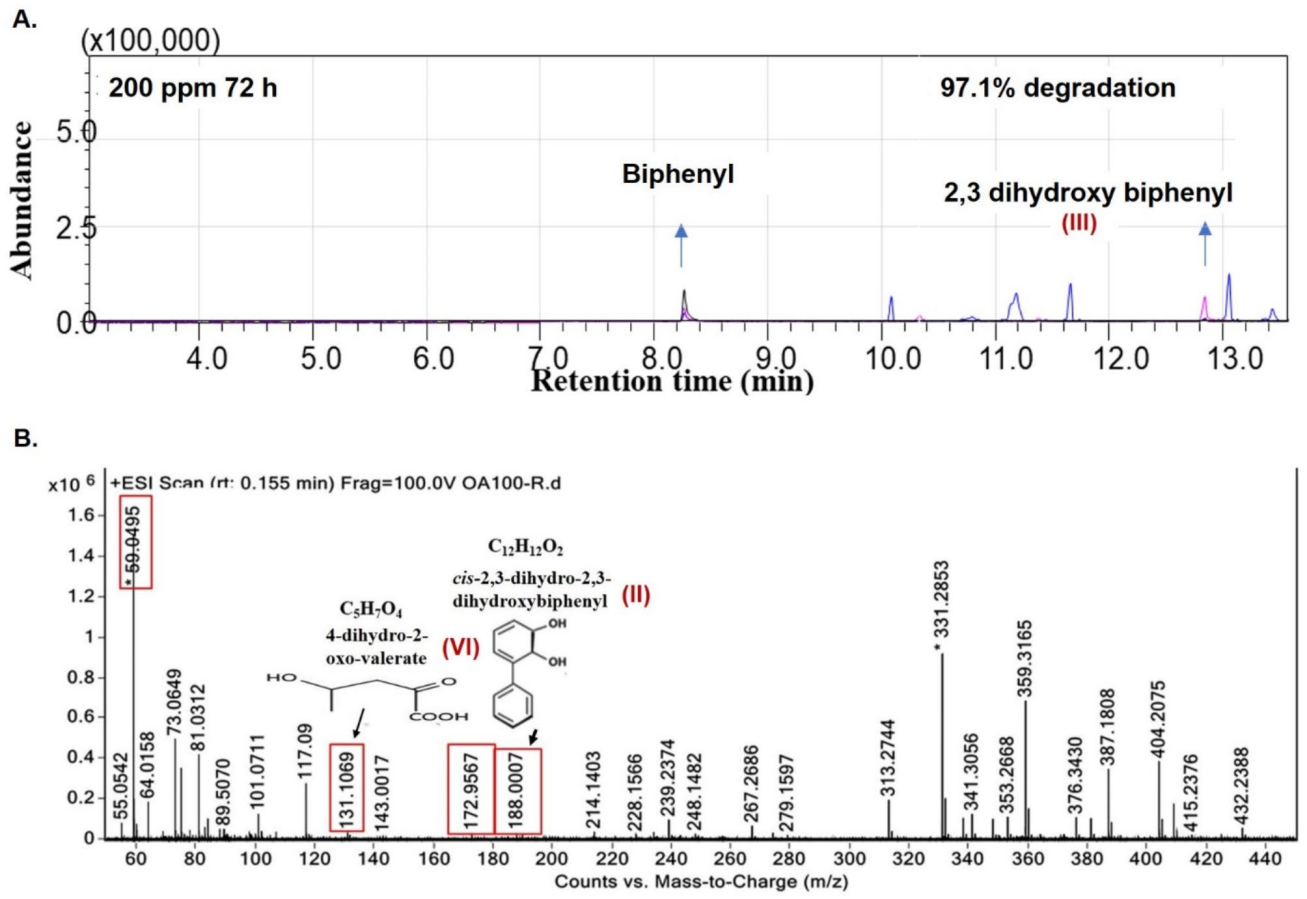
Metabolic product during the biphenyl degradation by MAPB-9 (**A**) GC-MS/MS identification of 2,3-DHB (1,1’ biphenyl 2,3-diol, III) by MRM mode (**B**) HR-MS identification of cis-2,3-dihydro-2,3-dihydroxybiphenyl (II), and 4-dihydro-2-oxo-valerate (VI). The identification of the metabolites (II) and (III) which confirms the presence of enzyme biphenyl 2,3-dioxygenase and cis-2,3-dihydro biphenyl-2,3-diol dehydrogenase, the two initial enzymes that resulted in biphenyl degradation. Further, the metabolite (VI) detection indicated complete mineralization *via* 4-hydroxy-2-oxovalerate aldolase to pyruvic acid that enters the TCA cycle.

Metabolites produced from biphenyl degradation by *B. anthropi* MAPB-9 were analyzed by GC-MS/MS and by HR-MS methods. Metabolites produced during the biphenyl degradation were extracted and analyzed by GC-MS/MS after the derivatization with BSTFA-TCMS. The GC-MS/MS mass spectra *via* MRM mode confirmed the presence of 2,3-dihydroxy biphenyl (1,1’ biphenyl 2,3-diol; 2,3 DHB) metabolite (**III)** in the extract from biphenyl degradation. (Fig. 2A). Similar results were obtained from the experiments with *Ochrobactrum anthropi*, now known as *B. anthropi*^17^, where 2,3 DHB formation was identified after 24 h. HRMS analysis of the extract resuspended in methanol confirmed the presence of cis-2,3-dihydro-2,3-dihydroxybiphenyl metabolite (**II)**, and 4-dihydro-2-oxo-valerate (also known as 4-Hydroxy-2-oxopentanoaic acid) metabolite (**VI)** with a well-resolved peak at R_t_ 1.55 min in ESI + mode scan with 188.007 and 131.1069, mass/charge ratio, respectively (Fig. 2B). Cis-2,3-dihydro-2,3-dihydroxybiphenyl is the intermediate of biphenyl formed by biphenyl 2,3-dioxygenase (BphA) and is converted to the second intermediate 2,3 DHB by cis-2,3-Dihydro-2,3-dihydroxybiphenyl dehydrogenase (BphB). In addition to the parent compound biphenyl, benzoate metabolite (**VII)**, and 2,3 dihydroxybenzoic acid metabolite (**IX)** were identified at 17.6 and 25.5 min, respectively, and are involved in the benzoic acid degradation pathway by *B. anthropi* MAPB-9 (Table 1).

Our results align with a description of the biphenyl degradation pathway as follows. The biphenyl degradation is initiated by the enzyme dioxygenase, which opens up the aromatic ring making it unstable. Biphenyl is converted to compound cis-2,3-dihydro-2,3-dihydroxy biphenyl (II), which is the first metabolite of the degradation process. Further, it is converted into 2,3-dihydroxy biphenyl (III) and 4-dihydro-2-oxo-valerate (VI). Various studies highlight that 4-dihydro-2-oxo-valerate is further degraded to pyruvate and acetaldehyde indicating complete degradation of biphenyl^2^.

**Table 1:** GC-MS/MS mass spectral features of the TMS-derived metabolites generated from biphenyl interconnected to the benzoic acid degradation pathway.

Metabolites	Structure	Rt	Mass spectra
Benzoic acid (VII)		17.6	194,179,135,105,77,51
2,3 dihydroxy benzoic acid (IX)		25.5	355,249,193,105,73,45

**Fig. 3:**
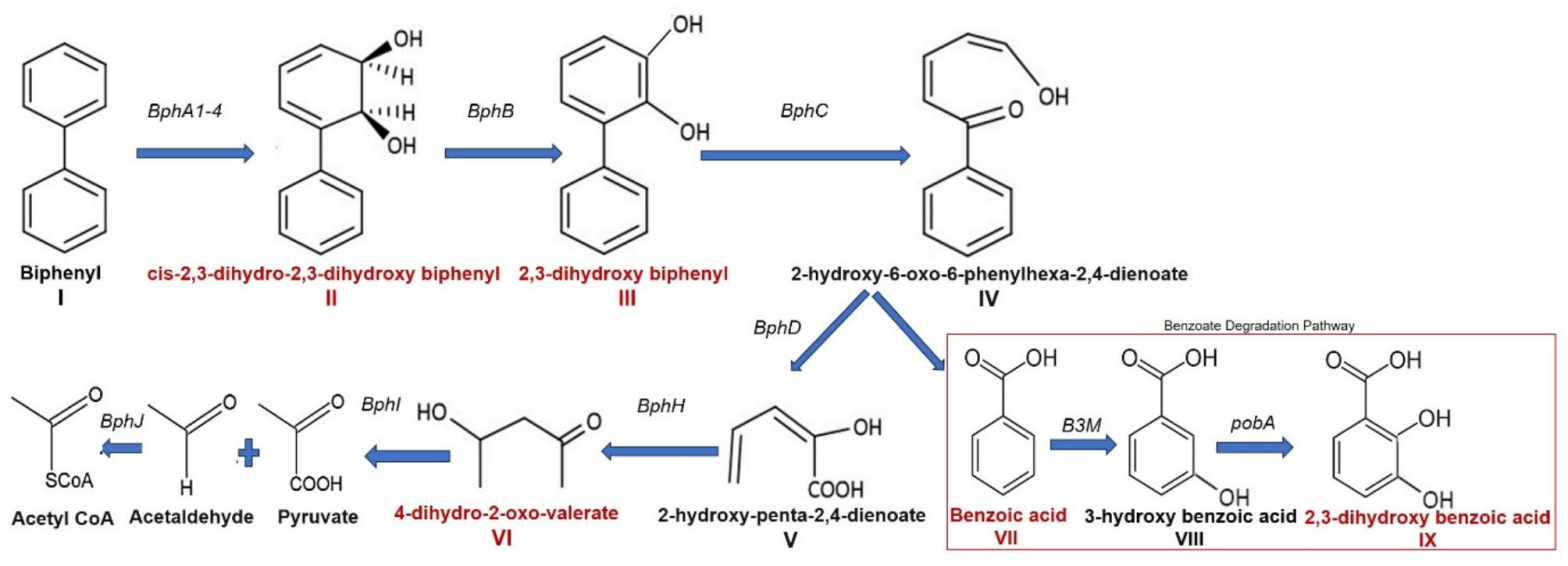
Complete biphenyl degradation pathway by *Brucella anthropi* MAPB-9 as confirmed by the identified metabolites produced during the degradation process. Biphenyl (**I**) degraded to *cis*-2,3-dihydro-2,3-dihydroxy biphenyl (**II**) followed by 2,3-dihydroxy biphenyl (**III**). Further metabolite (**III**) is degraded to 2-Hydroxy-6 oxo-6-phenyl-2,4-dienoate (**IV**). Metabolite (**IV**) forms two degraded products, 2-hydroxy-penta-2,4-dienoate (**V**) and Benzoic acid (**VII**). Metabolite (**V**) further degrades to 4-dihydro-2-oxo-valerate (**VI**) following a complete biphenyl degradation pathway. Benzoic acid (**VII**) is converted into 3-hydroxy benzoic acid (**VIII**) and further to 2,3-dihydroxy benzoic acid (**IX**) indicating the presence of genes involved in the benzoate degradation pathway. *cis*-2,3-dihydro-2,3-dihydroxy biphenyl (**II**) and 4-dihydro-2-oxo-valerate (**VI**) were identified by HRMS, while 2,3-dihydroxy biphenyl (**III**), Benzoic acid (**VII**) and 2,3-dihydroxy benzoic acid (**IX**) were identified by GC-MS/MS. The names of genes encoding enzymes catalyzing each reaction are shown on corresponding arrows. Genes involved *bph*A1A2A3A4, biphenyl 2,3-dioxygenase; *bphB*, cis-2,3-dihydro biphenyl-2,3-diol dehydrogenase; *bphC*, biphenyl-2,3-diol 1,2-dioxygenase; *bphD*, 2,6-dioxo-6-phenylhexa-3-enoate hydrolase; *bphI*, 2-hydroxypenta-2,4-dienoate hydratase; *bphI*, 4-hydroxy-2-oxovalerate aldolase; B3M, benzoate 3-monooxygenase; *pobA*, 4-hydroxybenzoate 3-monooxygenase. Metabolites in red are identified compounds in the extract of MAPB-9, while compounds represented in black are the other intermediates involved during the biphenyl degradation pathway.

Further, the identification of the benzoic acid (**VII**) followed by hydroxylated benzoic acid (2,3-hydroxybenzoic acid, **IX**) in the extracts reveals the occurrence of benzoic acid degradation by *meta-*cleavage pathway channeled into central metabolism. Benzoic acid catabolic pathways, such as the *β*-ketoadipate pathway, are widespread in the soil environment^18^. According to these results, the biphenyl and benzoic acid degradation pathways are found to be interconnected with the benzoic acid intermediate (Fig. 3). Moreover, these two metabolic pathways confirm the cleavage of the aromatic ring and the complete degradation of the biphenyl.

### Metabolites identified for carbon, nitrogen, and fatty acid metabolism formed by the selected bacterial isolates grown in biphenyl-supplemented MM

**Fig. 4:**
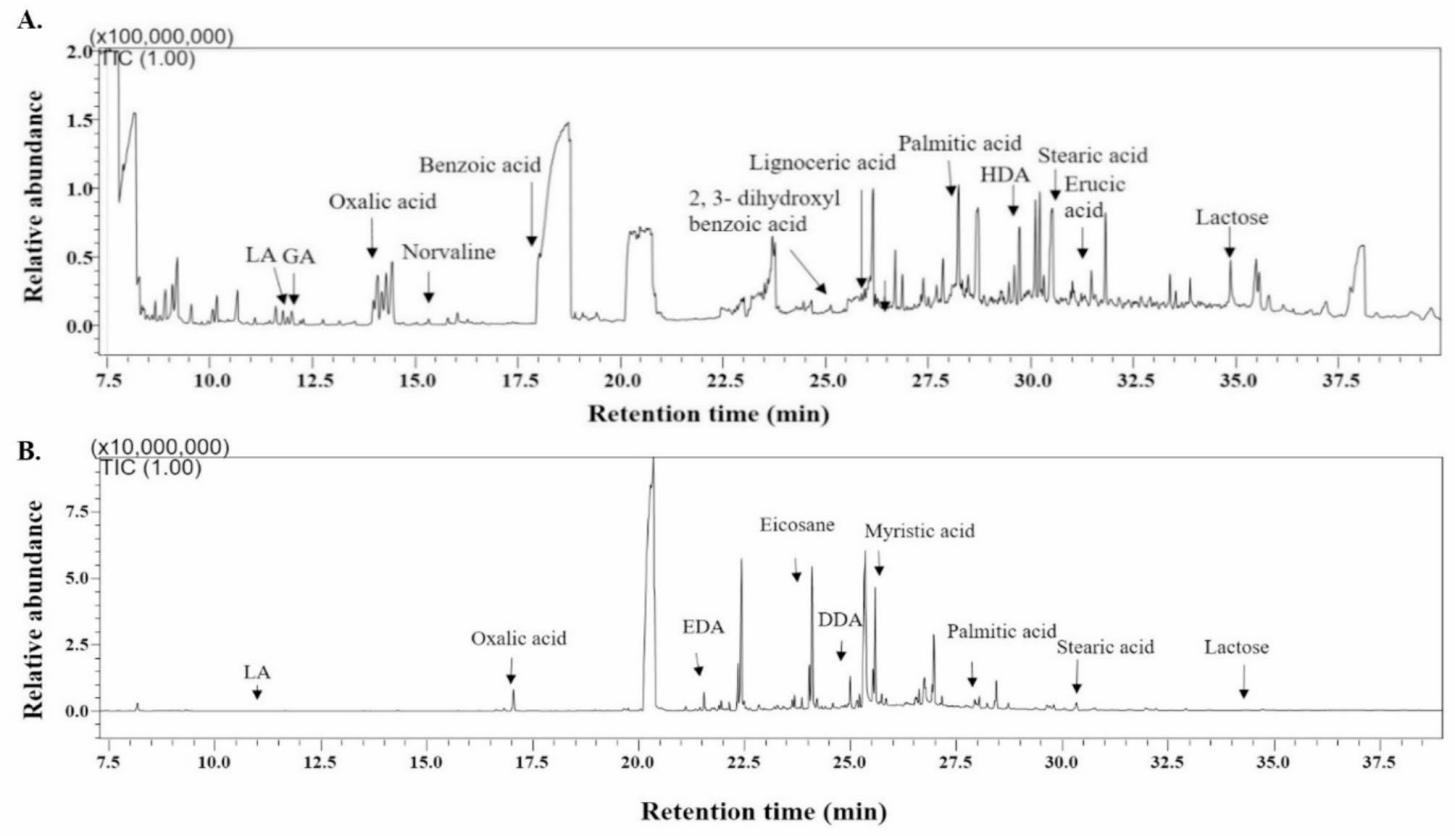
GC-MS/MS analysis of the metabolomic profile of the *Brucella anthropi* MAPB-9 grown in (**A**) biphenyl supplemented MM (**B**) 0.2% glucose in MM. LA, GA, EDA, DDA, and HAD represent lactic acid, glycolic acid, 11,14-eicosadienoic acid, dodecenoic acid, and heptadecanoic acid, respectively.

Since biphenyl is a non-natural source of carbon, the bacterial extract was further analyzed to identify metabolites and the metabolic pathways other than the biphenyl degradation pathway to investigate the effect of biphenyl on the metabolic activities of MAPB-9. The growth of MAPB-9 on biphenyl-supplemented MM indicated a direct effect on its metabolic activities resulting in different types of metabolites produced during the incubation period. In general, larger numbers of different metabolites were observed in biphenyl-grown bacterial isolate MAPB-9 than in their respective control. Different organic acid, fatty acid, sugar, and amino acid metabolites were identified by the mass spectral NIST14 library as shown in Fig. 4. The accumulation of organic acids such as oxalic, glycolic, lactic, and 1,4-butanediol in the media, are known metabolites formed during tricarboxylic acid (TCA) cycle, glyoxylate shunt, pyruvate metabolism, propanoate metabolism, amino acid biosynthesis, and fatty acid metabolism as described below.

### TCA, glyoxylate, and dicarboxylate metabolism

Glycolysis and TCA are the two important biochemical pathways necessary for cell maintenance and function. Organic pollutants such as biphenyl/PCB not only have adverse effects on these cellular pathways but also generate oxidative stress causing damage to different cell morphology, proteins, or fatty acid composition^19^.

GC-MS/MS analysis of the metabolites produced during biphenyl degradation by the MABP-9 grown on MM supplemented with 200 mg L^−1^ biphenyl for 72 h was conducted and showed significant fold change in metabolite abundance as compared to the control (Table 2). Significantly altered intracellular metabolites of *B. anthropi* MAPB-9 were observed, and the fold change showed increased metabolite levels from MAPB-9 with biphenyl stress. GC chromatograph represents the peaks of organic acids, fatty acids, and sugar, amino acids produced by MAPB-9 in biphenyl supplemented MM (Fig. 4 A) and in control with 0.2% glucose (Fig. 4B). Organic acid metabolites involved in the citric acid cycle were identified in the biphenyl-supplemented MM. Tricarboxylic acid cycle-related metabolites identified in the extract were citrate, isocitrate, and *cis*-aconitate. Aconitic acid and itaconic acid identified in MAPB-9 extract are intermediates of the tricarboxylic acid cycle.

**Table 2:** The identified metabolites of *Brucella anthropi* MAPB-9 showed a significant fold change with the biphenyl stressed condition compared to the glucose conditions. Compared to the non-stressed conditions, the fold change shows metabolite levels from MAPB-9 with biphenyl (200 Mg L^− 1^).

Sr. no.		Metabolites		log2 (FC)	Sr. no.			Metabolites	log2 (FC)
1		Oxalic acid		8.186	26			cis-13-Eicosenoic acid	2.3219
2		Palmitic Acid		7.563	27			Octadecane-1,2-diol	2.3219
3		Aconitic acid		4.5996	28			Methyl 3,4-dihydroxybenzoate	2.3219
4		Glycolic acid		4.5734	29			beta-D-Galactopyranoside	2.3219
5		D-(-)-Lactic acid		4.196	30			beta-Lactose	2.3219
6		Stearic acid		4.0998	31			L-Rhamnose	2.3219
7		Phenol		3.5029	32			Citronellic acid	-2.3219
8		Pentanoic acid		-3.412	33			Alanylthreonine	-2.3219
9		Decanoic acid		-2.6986	34			L-Fucose	-2.3219
10		1,4-Butanediol		-2.3731	35			Glucose	-2.3219
11		2-Ketobutyric acid		-2.3307	36			Glutamine	2.1022
12		Aceturic acid		2.3219	37			Benzoic Acid	2.0666
13		Methoxyacetic acid		2.3219	38			Maltose	1.9829
14		1-Butanol		2.3219	39			Hexanoic acid	1.9064
15		L-Norvaline		2.3219	40			beta-L-(-)-Fucopyranose	1.7583
16		Malic acid		-2.3219	41			13-Docosenoic acid, (Z)	-1.6115
17		Butanedioic acid		-2.3219	42			Citric acid	-1.5636
18		2-Octenoic acid		-2.3219	43			Heptanoic acid	1.3761
19		Oleic acid		2.3219	44			Pyruvic acid	1.3652
20		D-(-)-Citramalic acid		-2.3219	45			2,6-Bis(tert-butyl)phenol	-1.3259
21		2,3-Dihydroxybenzoic acid		2.3219	46			Stigmast-5-en-3-ol, oleate	-0.99721
22		Isovanillic acid		2.3219	47			Myristic acid	0.84309
23		Lignoceric acid		2.3219	48			Nonanoic acid	-0.76207
24		Hexadecanoic acid		2.3219	49			Pentadecanoic acid	0.64002
25		10-Heptadecenoic acid		2.3219					

The Aconitic acid showed a significant fold change in biphenyl-supplemented media as compared to the control, which showed its upregulation in the presence of biphenyl. Dehydration of citric acid results in the formation of *cis*-aconitic acid which transports to the cytosol^20^, where it is reported to be converted to itaconic acid by the enzyme *cis*-aconitic acid decarboxylase. The metabolites involved in the glyoxylate were also identified in the extract. The glyoxylate cycle and tricarboxylic acid cycle share five of the eight enzymes. The glyoxylate cycle bypasses the carbon dioxide-producing steps of the tricarboxylic acid cycle and is essential for acetate and fatty acid metabolism in bacteria. Several reports indicate that oxidative stress induces glyoxylate shunt in bacteria such as *P. aeruginosa*^21^. Bacteria such as *P. aeruginosa*, which utilizes acetate and fatty acids as sole carbon sources through the glyoxylate pathway, show a 10-fold increase in fluorescence by ROS generation when grown in biphenyl as the sole carbon source^19^. The above results also indicate that organic pollutants like PCB are reported to induce oxidative stress, which is supported by earlier reports^22^.

The glyoxylate shunt is up-regulated when acetyl-CoA is a direct product of a metabolic pathway, for example, *via* the degradation of acetate, fatty acids, and alkanes^23^. Metabolites such as acetate, glycine, succinate, citrate, glycolate, oxalate, *cis*-aconitate, (R, R)-tartaric acid, and ethylene glycol identified in the extract of MAPB-9 are well-known metabolites formed during the glyoxylate pathway. A higher accumulation of oxalic acid in the glyoxylate pathway was found in the biphenyl-supplemented culture (Fig. 4A) than in the control (Fig. 4B). Oxalic acid, and glycolic acid were significantly upregulated in stress-exposed cells as shown in Table 2. Higher accumulation of oxalates has also been reported in many bacteria under stress. Oxalate has been reported to chelate metals, which may help to alleviate oxidative stress^24^. Also, glycolate production is assumed to be the subject of bacterial oxidation mainly to conserve energy. Overall, the upregulation of aconitate, oxalate, and glycolate indicates significant alteration of metabolites by MAPB-9 to combat biphenyl stress. Hence, the glyoxalate pathway appears to result from a stress response induced by biphenyl.

### Salicyclic acid biosynthesis

Salicylic acid (SA) is a plant defense hormone that regulates various cellular processes. Many bacterial spp. also produce SA as an intermediate compound, which ultimately incorporates into salicylate-based siderophores. Salicylic acid is a secondary metabolite produced by most bacteria^25^, such as *Pseudomonas*, *Bacillus*, *Achromobacter*, and *Mycobacteria*^26,27^. The bacterial salicylate production is distinct from that of plants. Under an iron-deprived environment, salicylic acid biosynthesis is reported to be involved in the production of ferric-ion-chelating molecules, salicyl-derived siderophores catecholate^28^ especially in plant growth-promoting rhizobacteria (PGPR). Alkylated salicylic acid (6-[12(Z)-Nonadecenyl] salicylic acid), a derivative of salicylic acid, was found to be present in the extract of MAPB-9. SA has also been reported as an inducer of PCB degradation, and it has the potential to enhance the biodegradation of PCBs^29^. The salicylate catabolic gene cluster *bph* and *sal* gene are found to be cross-regulated in *P. pseudoalcaligenes* KF707, which are involved in the biphenyl degradation pathway^30^.

### Sugar moieties and monosaccharide metabolites in EPS

Extracellular polymeric substance (EPS) is the heterogeneous matrix of polymers that contain mainly long-chain polysaccharides, lipids, nucleic acids, and proteins^31^. EPS is synthesized intracellularly either during late logarithmic or stationary phase of growth. In the present study, EPS accumulated extracellularly on the cell surface as observed by FESEM analysis (Fig. 1). They are reported to protect the bacterial cells by stabilizing membrane structure and serve as carbon and energy reserves. Some sugar moieties and molecules, such as fucopyranose, cellobiose, melibiose, and lactose, were identified in the MAPB-9 extract. D-galactose is the hydrolysis product of lactose and is reported as an exopolysaccharide (EPS) component of the biofilm matrix produced by *Bacillus*^32^. Our observation is in agreement with the study of Onbasli and Aslim^33^, which reported high production of EPS by *P*. *aeruginosa* B1, *P. fluorescens* B5, *P. stutzeri* B11, and *P. putida* B15 grown in MM-supplemented with various organic pollutants (2,4-D, benzene, BTX, and gasoline) as sole carbon source. Several biosurfactant-producing bacteria, such as *Pseudomonas*, *Ochrobactrum*, and *Bacillus*, have been isolated from contaminated environments^34^. Other metabolites such as lactate, L-rhamnose, D-sorbitol, and L-fucose are known metabolites of fructose and mannose metabolism, which were also identified through GC-MS/MS analysis of MABP-9 extract. Lactate is the link between different metabolic pathways, as it is the substrate for gluconeogenesis and the product of glycolysis. While rhamnose and sorbitol are the sugars involved in biosurfactant formation. There are reports on the relation between the production of biosurfactants and the expression of the rhamnolipid synthesizing gene *rhl*AB. It has been reported to show a significant (*P* < 0.01) up-regulation of up to 258 folds on increasing biphenyl stress. Biphenyl-induced nutritional/environmental stress conditions highlighted enhanced bacterial rhamnolipid production, which, in turn, leads to higher solubilization efficiency of biphenyl in the bacterial cell. An increase in biphenyl concentration up-regulated *rhl*AB and *bph*A gene expression many folds in this bacterium^35^.

Also, EPS containing neutral sugars (glucose, galactose, and pyruvate) have been reported in *Pseudomonas* sp., such as *P. putida*, and *P. fluorescens*, when grown in an organic compound as a sole carbon source in MM^36^. Royan et al. demonstrated that *P. mendocina*, when grown in sodium benzoate as the sole carbon source, produces EPS content composed of rhamnose, fucose, glucose, ribose, arabinose, and mannose^37^. From the above discussion, it appears that bacteria produce EPS under stress conditions. Furthermore, the production of biosurfactants helps in the solubilization of biphenyl and PCBs, making them bioavailable for degradation^38,39^. Thus, EPS and biosurfactants identified in the MAPB-9 extract are reported to play an important role in the degradation of biphenyl and PCBs, further aiding in their bioremediation.

### Fermentation products

Fermentation is an anaerobic process in which bacteria use organic molecules as their final electron acceptor to produce fermentation end-products. Different fermentative products, such as lactic acid, malonic acid (propanedioic acid), 2,3-butanediol, acetoin, and butanedioic acid (succinic acid), were found in the extract of MAPB-9 grown in biphenyl-supplemented medium (Fig. 4A). Propanedioic acid (malonic acid) identified in the extract is a fermentative product of lactic acid. The selected bacteria is known to be facultative anaerobes; therefore, in the screw cap vials, there is a chance of low oxygen or anaerobic condition after some time.

### Amino acid metabolism

Amino acid accumulation is considered to be one of the stress responses in bacteria^40^. The amino acid accumulation could be either for the synthesis of new protective proteins or due to increased protein degradation. Glutamine was found to be significantly upregulated in biphenyl-exposed cells, as shown in Table 2; Fig. 6. Glutamate and the derivatives of this amino acid play essential roles in nitrogen metabolism and stress tolerance. The glutamate-dependent acid tolerance system has been reported as critical for bacteria to survive in acidic environments. Glutamine converts into glutamate in the cytoplasm by acid-activated YbaS, and initiates the glutamate decarboxylase (GAD) system. The formation of alkaline products (ammonia and GABA) and the reduction of intracellular protons are the net consequences of this glutamate-related metabolism^41^.

### Fatty acid metabolism

Fatty acids are essential components of membranes and are important sources of metabolic energy in all organisms. Biphenyl-induced stress increases saturation and changes in branched fatty acids in total lipids (TL). Alteration in the fatty acid composition of membrane lipids is one of the critical adaptation mechanisms of bacteria against organic compounds such as biphenyl. Bacteria undergo modification in the cell membrane as an adaption mechanism for survival. Membrane stress responses modify the cell membrane by modulating the length, branching, and saturation of the fatty acid acyl chains, altering membrane lipid composition, and synthesizing proteins that modify or protect the membrane^42^. In our study, the fatty acid profile showed the presence of lignoceric acid, palmitic acid, 9-octadecenoic acid, (E), stearic acid, 13-docosenoic acid at R_t_ 26.5, 28.7, 30.3, 30.5, and 31.2 min, respectively (Fig. 4A). The TMS derivative stearic acid, palmitic, 11,14-eicosadienoic acid, and 5-dodecenoic acid (Z) were found in the extract of MAPB-9, showing a high percentage of saturated fatty acid in the total lipid (Fig. 4A) than in control (Fig. 4B). Principal component analysis (PCA) of the fatty acid metabolite showed a relatively higher abundance of saturated fatty acids in the presence of biphenyl as compared to the control in the experimental sets.

**Fig. 5:**
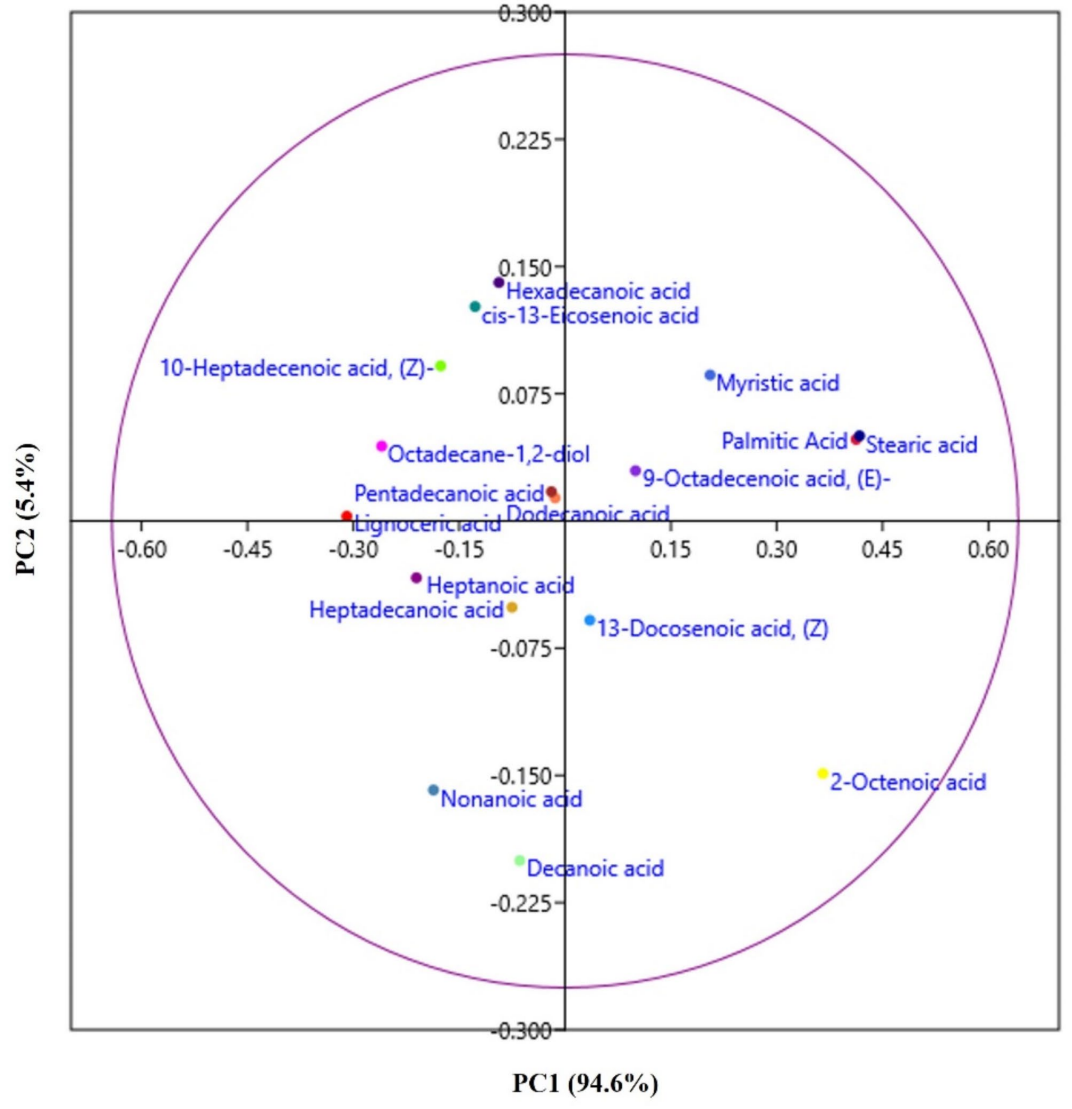
Principal component analysis of the metabolite fatty acid profile of MAPB-9 grown in MM supplemented with biphenyl and control. The percentage of total variance represented by principal component 1 (PC1) and principal component 2 (PC2) are shown in parentheses.

The total lipid comprised high levels of palmitic (C16:0), stearic acid (C18:0), Myristic, 9-octadecenoic acid content in biphenyl-treated MAPB-9, as shown in Fig. 5. Previous studies suggest that increased levels of stearic and palmitic acid, a fully saturated fatty acid, results in the stiffening of the cell membrane, thereby reduces its permeability to small molecules under stress^43^. An increase in saturated fatty acids rather than unsaturated has been reported to be the most common adaptive response in the metabolism of toxic pollutants^44^. The possible reason for the relatively high abundance of saturated fatty acid identified in the MAPB-9 extracts could be cell adaptation, including the bioavailability of the biphenyl to the bacterial cell for degradation. Therefore, the accumulation of fatty acids in the selected bacterial isolate extracts indicated a metabolic strategy to combat biphenyl-induced nutritional stress.

### Metabolite set enrichment analysis

**Fig. 6:**
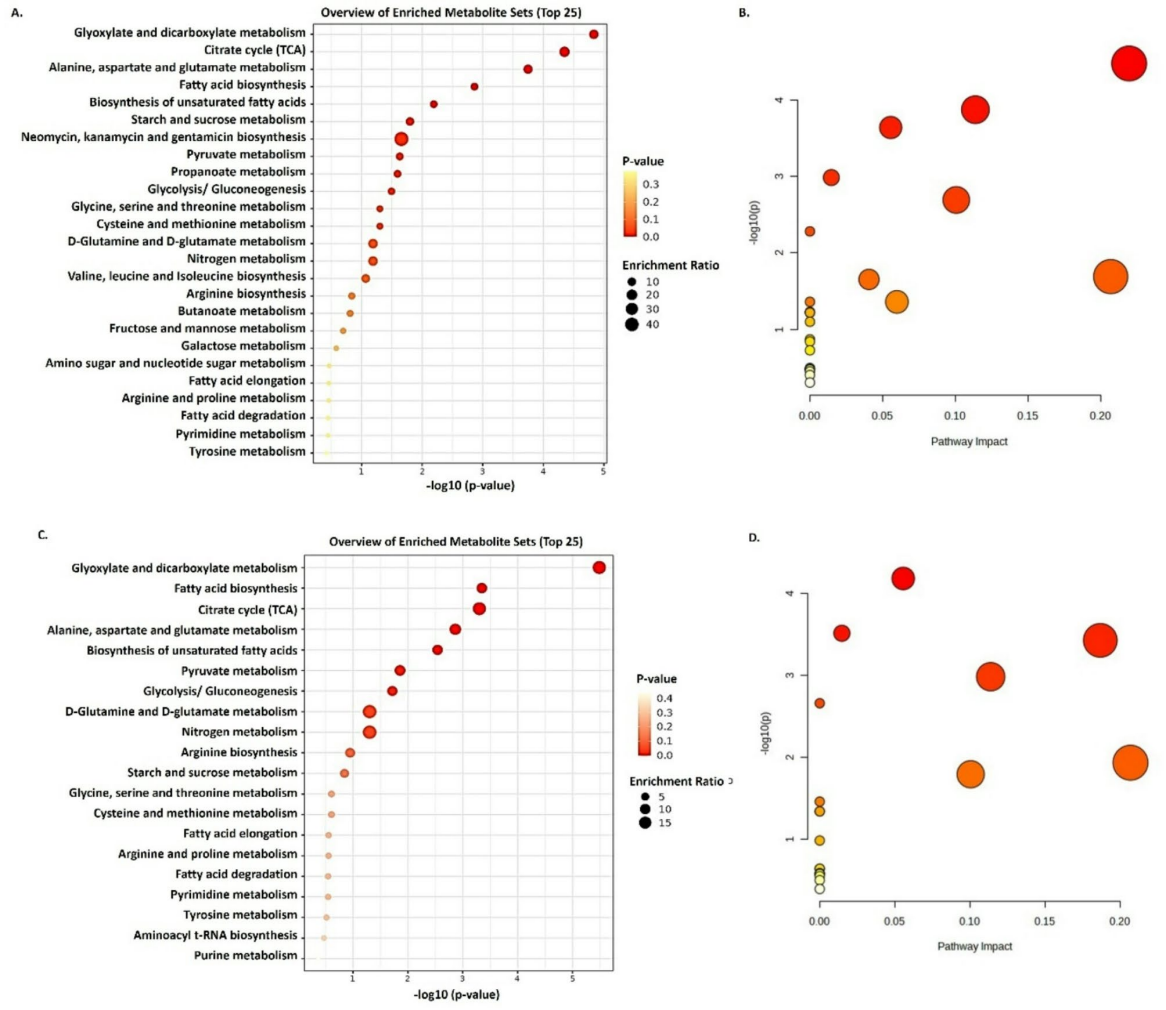
Metabolite Set Enrichment Analysis to identify biologically meaningful patterns significantly enriched in metabolomic data. Metabolomic data analysis was done by using MetaboAnalyst 5.0 software. The metabolites detected by the GC-MS/MS analysis were used as input for the analysis to determine the metabolic pathway in MAPB-9 under control (**A**,**B**) and biphenyl-induced stress condition (**C**,**D**). The enrichment ratio is computed by Hits / Expected, where hits = observed hits; expected = expected hits. Fatty acid biosynthesis and biosynthesis of unsaturated fatty acid metabolism were identified as the most prominent metabolism in MAPB-9. The larger the bubble, the greater the number of differential metabolites within the entry. The bubble color represents the P-value. The smaller the enrichment P-value, the greater its significance.

Metabolite enrichment analysis revealed the functions and involvement of these metabolites in various metabolic pathways. The enrichment analysis of the differential metabolite pathway was performed based on the KEGG database. Enrichment metabolite analysis (Fig. 6), which shows the top-25 bubble diagram of differential metabolites wherein the Rich Factor is the enrichment factor. The two main enriched metabolic pathways out of 25 metabolisms were glyoxylate and dicarboxylate metabolism and fatty acid metabolism in MAPB-9 during the biphenyl-stressed condition. Our results demonstrate that the TCA cycle, glyoxylate, dicarboxylate metabolism, alanine, aspartate and glutamate metabolism, and fatty acid biosynthesis metabolic pathways are significantly more active in response to biphenyl stress in *B. anthropi* MAPB-9 (Fig. 6).

Fatty acid biosynthesis metabolism is interconnected with the glyoxylate pathway. A previous report suggests that PCB accumulation results in increased fatty acid saturation of bacterial membranes^7^. Fatty acid biosynthesis is found to be the most active metabolism in MAPB-9. The biosynthesis of saturated fatty acids is reportedly linked to the membrane fluidity in bacteria such as *R. eutropha H850* cells under 2,2’,5,5’-tetrachlorobiphenyl and biphenyl environmental stress conditions^45^. Further, fatty acid degradation releases free acetyl-CoA, which can be reused for energy production via the TCA cycle or consumed to synthesize fatty acid. Thus, fatty acid degradation and biosynthesis pathways must be switched on and off according to the availability of fatty acids to maintain membrane lipid homeostasis.

When higher fatty acids are oxidized into acetyl-CoA without forming pyruvate, acetyl-CoA enters the glyoxylate cycle. Acetyl-CoA produced from acetate or by *β*-oxidation of fatty acids enters the TCA cycle. It condenses with oxaloacetate to form citrate, which further isomerizes to isocitrate, wherein isocitrate cleaves by isocitrate lyase to form glyoxylate and succinic acid. Succinic acid then metabolizes in the TCA cycle, while glyoxylate condenses with a second molecule of acetyl-CoA to form malate^46^.

**Fig. 7:**
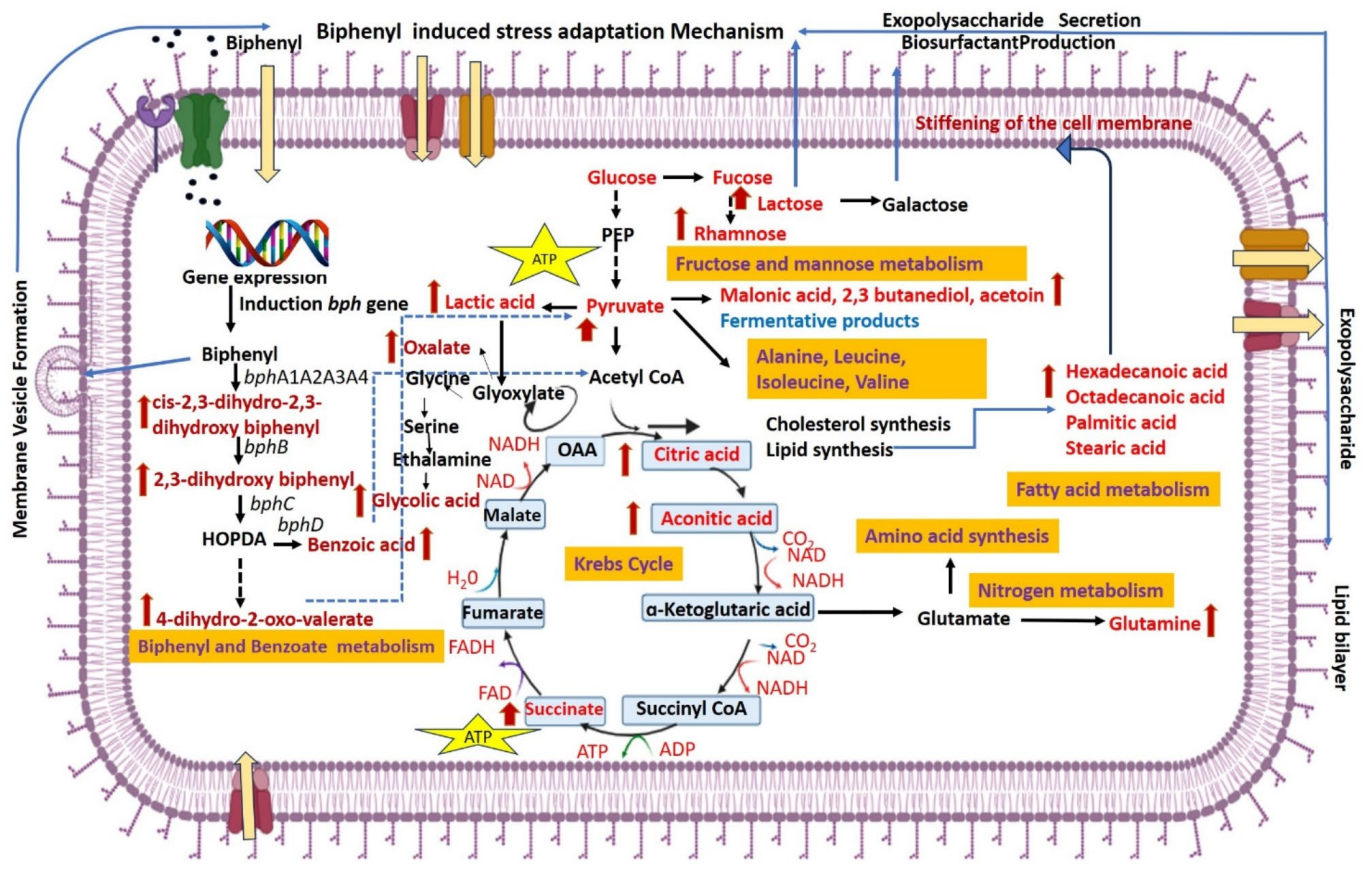
Overview of the metabolomic profile of the *Brucella anthropi* MAPB-9 exposed to biphenyl. The red-colored compounds shown in the image represent metabolites formed or upregulated during the biphenyl degradation pathway. The red arrow indicates the high level of metabolite in the extract of MAPB-9 supplemented with biphenyl. In the presence of biphenyl, the induction of biphenyl degrading enzyme leads to the accumulation and identification of metabolites that are converted to pyruvic acid *via* 4-hydroxy-2-oxovalerate aldolase. The pyruvic acid leads to the formation of acetyl CoA, which enters the TCA cycle and glyoxylate cycle. Glyoxylate pathway metabolites such as oxalate and glycolic acid were identified to be upregulated to conserve energy. The presence of lactic acid, malonic, 2,3 butanediol, and acetoin indicated the occurrence of the fermentation. Some metabolites found in the composition of exopolysaccharide and biosurfactant were also identified, showing the biphenyl-induced cell’s adaptive behavior.

Therefore, the observation highlights that MAPB-9 grown in the biphenyl-supplemented MM combat stress (nutritional stress) *via* the glyoxylate pathway to provide cellular precursors from one available carbon source and by converting biphenyl into intermediates that enter the TCA cycle In the presence of biphenyl, the induction of biphenyl degrading enzyme leads to the accumulation and identification of metabolites (1,1’ biphenyl 2,3-diol, cis-2,3-dihydro-2,3-dihydroxybiphenyl, 4-dihydro-2-oxo-valerate, and benzoic acid in the media extract. Further, the metabolite 4-dihydro-2-oxo-valerate identified is converted to pyruvic acid *via* 4-hydroxy-2-oxovalerate aldolase. The pyruvic acid leads to the formation of acetyl CoA, which enters the TCA cycle and glyoxylate cycle (Fig. 7). It has been reported that the evolution of metabolic pathways helps to regulate cellular function in response to nutrition and environmental stress.

## Conclusion

Overall, the cell morphology, metabolomic profiling results, and the metabolism enrichment analysis indicate that the *Brucella anthropi* MAPB-9 shows an adaptative mechanism to survive under biphenyl-induced stress conditions and utilize it as a carbon source. We conclude that the identification of the metabolites produced in the extract highlighted the active mechanism of the biphenyl degradation pathway. The biphenyl induces synthesis of biphenyl degrading enzymes, which catabolize biphenyl the former into intermediates such as 1,1’ biphenyl 2,3-diol, cis-2,3-dihydro-2,3-dihydroxybiphenyl, 4-dihydro-2-oxo-valerate, and benzoic acid. Further, 4-dihydro-2-oxo-valerate and benzoic acid enter the TCA cycle *via* pyruvate, indicating its complete mineralization. In addition, the upregulation of glyoxylate and dicarboxylate, as well as fatty acid biosynthesis metabolic pathways, was observed in response to biphenyl-induced stress in MAPB-9. Therefore, the present study highlights an understanding of the metabolomic and cellular processes that predicted the survival and activity of *B. anthropi* MAPB-9 exposed to biphenyl.

## Data Availability

Data will be available from the corresponding author upon reasonable request.
